# Performance improvements of virtual monoenergetic images in photon-counting detector CT compared with dual source dual-energy CT: Fourier-based assessment

**DOI:** 10.1007/s13246-024-01499-6

**Published:** 2024-12-10

**Authors:** Hiroki Kawashima, Katsuhiro Ichikawa, Ryoichi Yoshida, Takuto Katayama, Makoto Arimoto, Jun Kataoka, Hiroji Nagata, Satoshi Kobayashi

**Affiliations:** 1 Faculty of Health Sciences, Institute of Medical, Pharmaceutical and Health Sciences, Kanazawa University, 5-11- 80 Kodatsuno, Kanazawa, 920-0942 Japan; 2 Department of Radiology, Tokai University Hospital, 143 Shimokasuya, Isehara, 259-1193 Japan; 3 Faculty of Mathematics and Physics/Advanced Research Center for Space Science and Technology, Institute of Science and Engineering, Kanazawa University, Kakuma, Kanazawa 20-1192 Japan; 4 Faculty of Science and Engineering, Waseda University, 3-4-1 Okubo, Shinjuku-ku, Tokyo, 169-8555 Japan; 5 Section of Radiological Technology, Department of Medical Technology, Kanazawa Medical University Hospital, Daigaku 1-1, Uchinada, Kahoku 920-0293 Japan; 6 Department of Radiology, Kanazawa University Graduate School of Medical Science, 13-1 Takara-machi, Kanazawa, 920-8640 Japan

**Keywords:** Computed tomography CT, Photon-counting detector, Photon-counting CT, Image quality

## Abstract

To confirm the performance improvement of virtual monoenergetic images (VMIs) for iodine contrast tasks in a clinical photon-counting detector CT (PCD CT) using Fourier-based assessment, compared with those in the latest-generation dual-source dual-energy CT (DECT). A water-filled bath with a diameter of 300 mm, which contains rod-shaped phantoms equivalent to diluted iodine (2 and 12 mg/mL), was scanned using PCD CT and DECT at 15, 7.5, and 3 mGy. VMIs were generated without any iterative reconstruction algorithm. Task transfer function (TTF), noise power spectrum (NPS), and slice sensitivity profile were evaluated for VMIs at 70 and 40 keV. The detectability index (*d’*) and the squared system performance function (SPF^2^) calculated by TTF^2^/NPS were compared. At 40 keV, the *d’* values of PCD CT were higher (percentage increase of 25.7-39.9%) than those of DECT, whereas at 70 keV, the difference was rather small. The SPF^2^ values at 40 keV of PCD CT grew notably higher than those of DECT as the spatial frequency increased. The higher SPF^2^ values endorsed the lower image noise and the sharper edge of the rod phantom as observed. The *d’* and SPF^2^ in VMIs at 40 keV of PCD CT were notably higher than those of DECT, which endorsed the clinical advantages of PCD CT that had been previously reported in various studies.

## Introduction

In recent years, CT systems with a cadmium telluride-based photon-counting detector (PCD) have become available for patient imaging. PCD CT has reportedly several advantages over conventional CT systems with an energy-integrating detector (EID) [1–10]. In an iodine contrast-enhanced examination, the improved contrast-to-noise ratio (CNR) is one of the key benefits of PCD CT. This is thanks to the differences in the X-ray detection process between the EID and the PCD. With the EID, the intensity of the scintillation light (the detect signals) is proportional to the photon energy; therefore, low-energy photons are integrated with low weights. On the other hand, with the PCD, individual photons are counted and assigned to energy bins according to their energy [10]. No weighting is applied across different energy levels of the photon [1, 9, 10]. Thus, the contribution of low-energy photons in forming contrast information is greater with PCD CT than with EID CT. Furthermore, by introducing the lower threshold energy, electronic noise can be minimized in the data acquisition process of the PCD [2, 3]. Thus, the PCD CT is effective in improving image quality [1, 9, 10].

The PCD CT system can acquire multi-energy data. Virtual monoenergetic images (VMIs) are reconstructed by raw-data based analysis and mainly used in routine clinical practice. The properties of VMIs obtained via dual-energy acquisition using EID (DECT) have been evaluated by a number of previous studies. It is now well known that the image properties such as noise and contrast can be optimized by carefully selecting the energy level of the VMI in accordance with the examination needs [11, 12]. VMIs at 65–75 keV have relatively less noise and can be used as an alternative to images that are obtained with a standard tube voltage of 120 kV [13, 14]. Meanwhile, VMIs at low keV levels have the potential to show lesions more distinctly in contrast-enhanced CT scans, as well as low tube voltage techniques [15–17]. In particular, VMIs at 40 keV can visualize iodinated vessels and regions in organs in much higher contrast because the energy level is closer to the K-edge of iodine at 33 keV than at low tube voltages of 80 to 100 kV. As a result, it can even serve as a compensation for poor contrast enhancement in certain examinations [17]. However, in DECT, it should be noted that it is quite tricky to process low-energy VMIs in order to suppress the increase in noise resulting from the reconstruction for the low energy. In this processing, the edge information (i.e., high spatial frequency components) is brought from a mixture of high- and low-energy images (a low noise image) and is mixed with the contrast information (i.e., low spatial frequency components) of the low keV image [12, 18, 19]. For this matter, in PCD CT, a similar technique might be applied to reduce noise in the low keV image. Because of such processing, it would be better to consider the frequency characteristics in evaluating imaging performance than to use such a simple measure as CNR.

There are reports indicating that clinical PCD CT is advantageous in improving the imaging performance of VMIs [1–10]. Some researchers have evaluated the frequency-dependent performance of VMIs focusing on clinical use [20, 21]; however, the inherent performance of clinical PCD CT remains uncertain in some aspects. In particular, it is worthwhile to confirm the impact of the equal weighting of all photons in the data acquisition of the PCD on the frequency characteristics of VMI at 40 keV. Therefore, the purpose of this study is to evaluate the performance of VMIs in PCD CT compared with that in DECT, using Fourier-based assessment for detailed comparison.

## Methods

### Phantoms

**Fig. 1:**
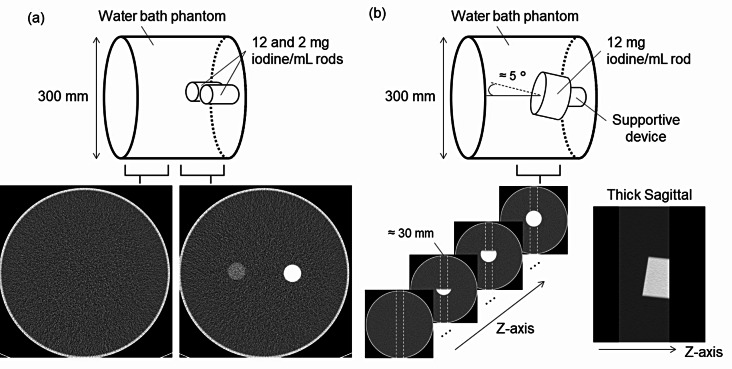
Experimental setup and sample CT images. **a** Image noise and in-plane resolution were measured in the uniform water section and the two-rod section. **b** Slice sensitivity profile was measured from a slant edge plane in a thick sagittal image. The sagittal image with a thickness of approximately 30 mm was created from axial images

A base phantom was a water-filled bath with a diameter of 300 mm, replicating the adult abdomen from the viewpoint of X-ray absorption. For the in-plane resolution measurements, two cylindrical rods, 30 mm in diameter, made of materials equal to diluted iodine with 12 and 2 mg iodine (mgI)/mL (Kyoto-Kagaku) were used. These rods were placed in parallel to the rotation axis of the CT system, 50 mm offset from the center of the base phantom (Fig. 1a). For the slice sensitivity profile (SSP) measurements, we replaced the two rods with one rod, 70 mm in diameter, made of the same material with 12 mgI/mL, which was placed at the center of the base phantom. This rod was slightly angled by approximately 5 degrees with respect to the image plane [22] and the bottom of the rod using a supportive device (Fig. 1b).

## Data acquisition

The phantom scanning was performed with two CT scanners: a first-generation PCD CT scanner (NAEOTOM Alpha, Siemens Healthineers) and a latest-generation dual-source CT scanner (DECT; SOMATOM Force, Siemens Healthineers). The scan and reconstruction parameters are summarized in Table 1. The scan parameters were determined based on the abdominal imaging protocol used for each scanner in the institute where the measurements were conducted. A tube voltage of 120 kV was used in PCD CT, whereas a combination of 80 and 150 kV with a tin filter (80/Sn150 kV) was used in DECT. The selected tube voltage and its combination were practical for the clinical use of the abdominal examination [23, 24]. Three CT dose indices of 15, 7.5, and 3 mGy were tested to assess the dose dependency on a standard- to low-dose CT protocol of the imaging performance [25, 26]. These CTDI values were ensured by adjusting tube current settings in each CT scanner without using any tube current modulation.

**Table 1:** Scan and reconstruction parameters

Scanner model	NAEOTOM Alpha PCD-CT	SOMATOM Force DECT
Tube voltage (kV)	120	80/Sn150
Rotation speed (s/rotation)	0.5	0.5
Pitch	0.8	0.8
Detector configuration (mm)	144 × 0.4	192 × 0.6
CT dose index (mGy)	15, 7.5, 3	15, 7.5, 3
Slice thickness (mm)	1.0	1.0
Display field of view (mm)	300	300
Matrix size	512 × 512	512 × 512
Reconstruction	Qr44f (without iterative reconstruction)	Qr44d (without iterative reconstruction)
Energy level for VMIs (keV)	70, 40	70, 40

PCD-CT, photon-counting detector CT; DECT, dual-energy CT; VMI, virtual monoenergetic image

VMIs of 70 and 40 keV were generated from each CT scanner with a slice thickness of 1.0 mm without a slice gap. The choice of these energy levels is because of their importance in clinical practice, as mentioned in the Introduction. The reconstruction kernel (Qr44) of the same name was used. We did not use any iterative reconstruction (IR) because different IR techniques were implemented in each scanner, and the differences may not be appropriate for comparing the inherent performance of PCD CT and DECT. For the data of SSP measurements, the minimum slice gap was set at 0.2 and 0.1 mm for PCD CT and DECT, respectively. The slice gap of 0.2 mm satisfies the correct detection of SSP with the slice thickness of 1.0 mm according to the sampling theorem.

## Image analysis

### In-plane resolution

The task transfer function (TTF) was measured from the images in the two-rod section (Fig. 1a), as in the previous reports [22, 27]. An edge spread function (ESF) was generated by using the circular edge technique. To ensure a CNR of more than 20 and obtain an ESF with sufficiently low noise, we averaged the rods’ images obtained by repeatedly scanning 5, 8, and 12 times for 15, 7.5, and 3 mGy, respectively, in PCD CT, and 6, 11, and 14 times for 15, 7.5, and 3 mGy, respectively, in DECT. The derivative of the ESF provided the line spread function. Then, the TTF was calculated from a discrete Fourier transform of the line spread function.

## Noise property

The noise power spectrum (NPS) was measured from the images in the water section. Four regions of interest (ROIs), each with 128 × 128 pixels, were set concentrically at a distance of 50 mm. The NPS was calculated based on an established method using a two-dimensional Fourier transform in each ROI [22, 28, 29]. A one-dimensional NPS was obtained by radially re-binning the two-dimensional NPS. The NPS results were averaged across all the ROIs of 50 consecutive images.

### Slice sensitivity

The SSP was measured using the edge surface of the approximately 30-mm thick sagittal images reformatted from an axial image set as shown in Fig. 1b [22, 30]. Multiple thick sagittal images obtained in 2–8 scans were averaged to reduce the effects of image noise. A synthetic edge profile was created using the edge method established in previous studies [22, 31, 32], and the derived edge spread function was differentiated to yield the SSP.

A direct comparison of image noise between PCD CT and DECT would not produce reasonable results, if the effective slice thickness differs between the two. Therefore, to compensate for the variances resulting from differences in effective slice thickness, we performed NPS correction by utilizing the inverse square relationship between image noise and slice thickness [33, 34]. The full width at half maximum (FWHM) of SSP was used as the index of the slice thickness.

## Detectability index and system performance function

The detectability index (*d’*) and the system performance function (SPF), which were calculated using the measured TTF and NPS, were used for imaging performance comparison of the VMIs between PCD CT and DECT. The *d’* value was computed by

$$\:{{d}^{{\prime\:}}}^{2}=2\pi\:\:\int\:f\:\frac{{TTF}^{2}\left(f\right)}{{NPS}_{c}\left(f\right)}\:{W}^{2}\left(f\right)\:df$$,

where *f* denotes the spatial frequency assuming radial symmetry, as in the calculation of the *d’* value with the ideal observer model [29, 35]. Although *NPS*(*f*) was used in the common expression of this observer model, we replaced it with its modified version [*NPS*_*c*_(*f*)] accounting for the slice thickness difference (NPS × correction factor). The slice thickness of PCD CT was taken as the reference (the correction factor of 1.0); the correction factor of DECT was the ratio of the FWHM of SSP for PCD CT to that of DECT. *W*(*f*) was defined as the Fourier transform of the object to be detected here,

$$\:W\left(f\right)=\frac{\pi\:}{2}{|\varDelta\:HU|d}^{2}\frac{{J}_{1}\left(\pi\:df\right)}{\pi\:df}$$,

with *J*_1_ being a first-order Bessel function and *d* being the disk diameter. In this study, we simulated tasks of a 5.0-mm diameter with an iodine contrast of 12 and 2 mgI/mL. The difference in CT number between the iodine rod and the water background (*ΔHU*) was obtained from the images at the two-rod section using a square region of interest of 25 × 25 pixels. The measurement results of 10 consecutive images were averaged.

In addition to *d’*, we calculated the system performance function {SPF(*f*)} given by TTF^2^(*f*)/NPSc(*f*), from which imaging performance over the entire spatial frequency can be evaluated [30, 35–38].

## Results

### Task transfer function

**Fig. 2:**
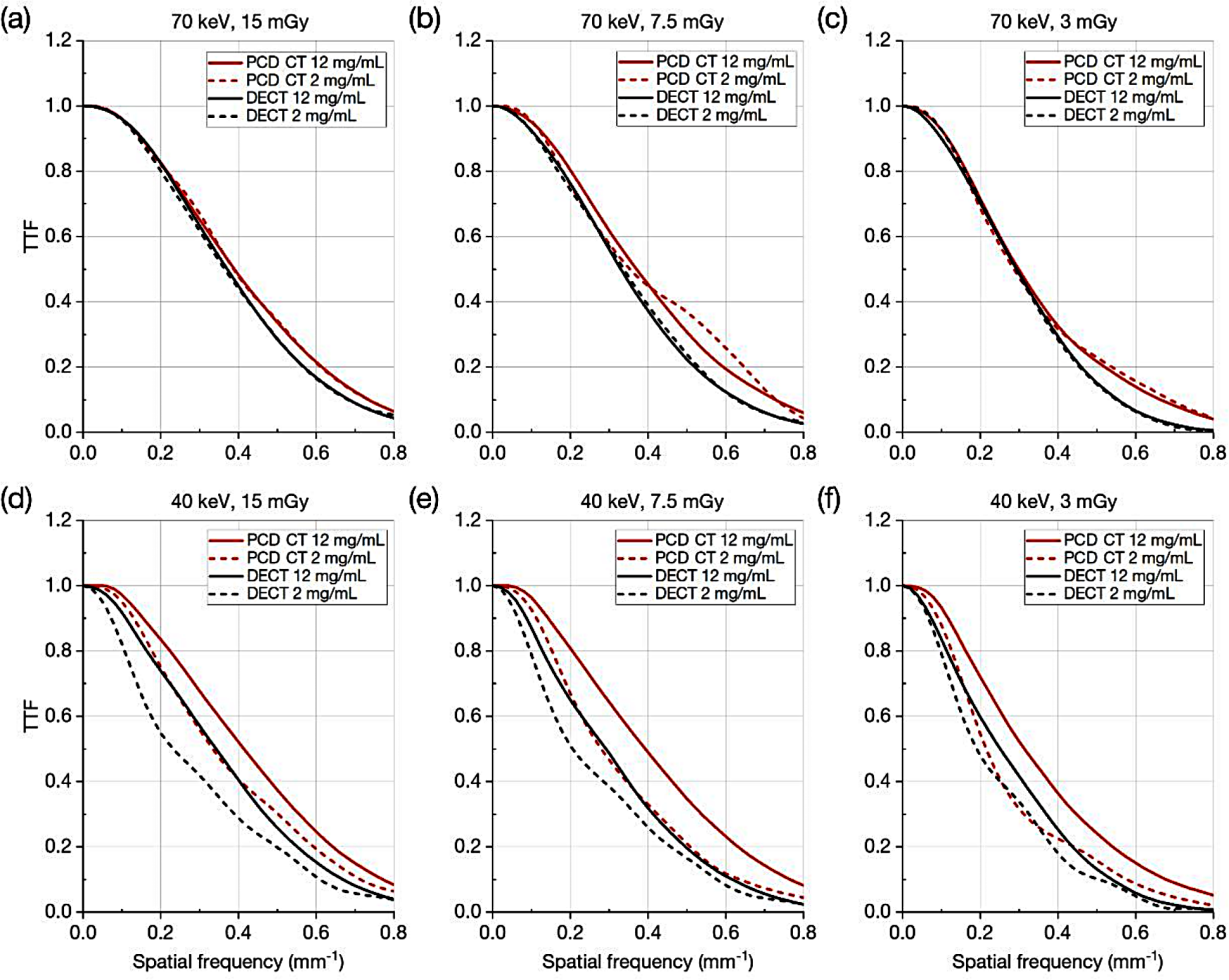
Comparison of task transfer function for 12 and 2 mg iodine/mL between photon-counting detector and dual energy CT. **a**, **b**, **c** 70 keV and **d**, **e**, **f** 40 keV at the volume CT dose indexes of **a**, **d** 15 mGy, **b**, **e** 7.5 mGy and **c**, **f** 3 mGy

The TTF results of PCD CT and DECT for a 12 mgI/mL and a 2 mgI/mL rods are shown in Fig. 2. At 70 keV, the TTF values of PCD CT were comparable to or somewhat higher than those of DECT. At 40 keV, the TTF values of PCD CT were notably higher than those of DECT. For example, the 50% TTFs for 12 mgI/mL of PCD CT and DECT were 0.41 and 0.34 mm^− 1^ at 15 mGy, 0.39 and 0.29 mm^− 1^ at 7.5 mGy, and 0.31 and 0.25 mm^− 1^ at 3 mGy. Also, the TTF values for 2 mgI/mL were notably lower than those for 12 mgI/mL in both PCD CT and DECT.

### Noise power spectrum

**Fig. 3:**
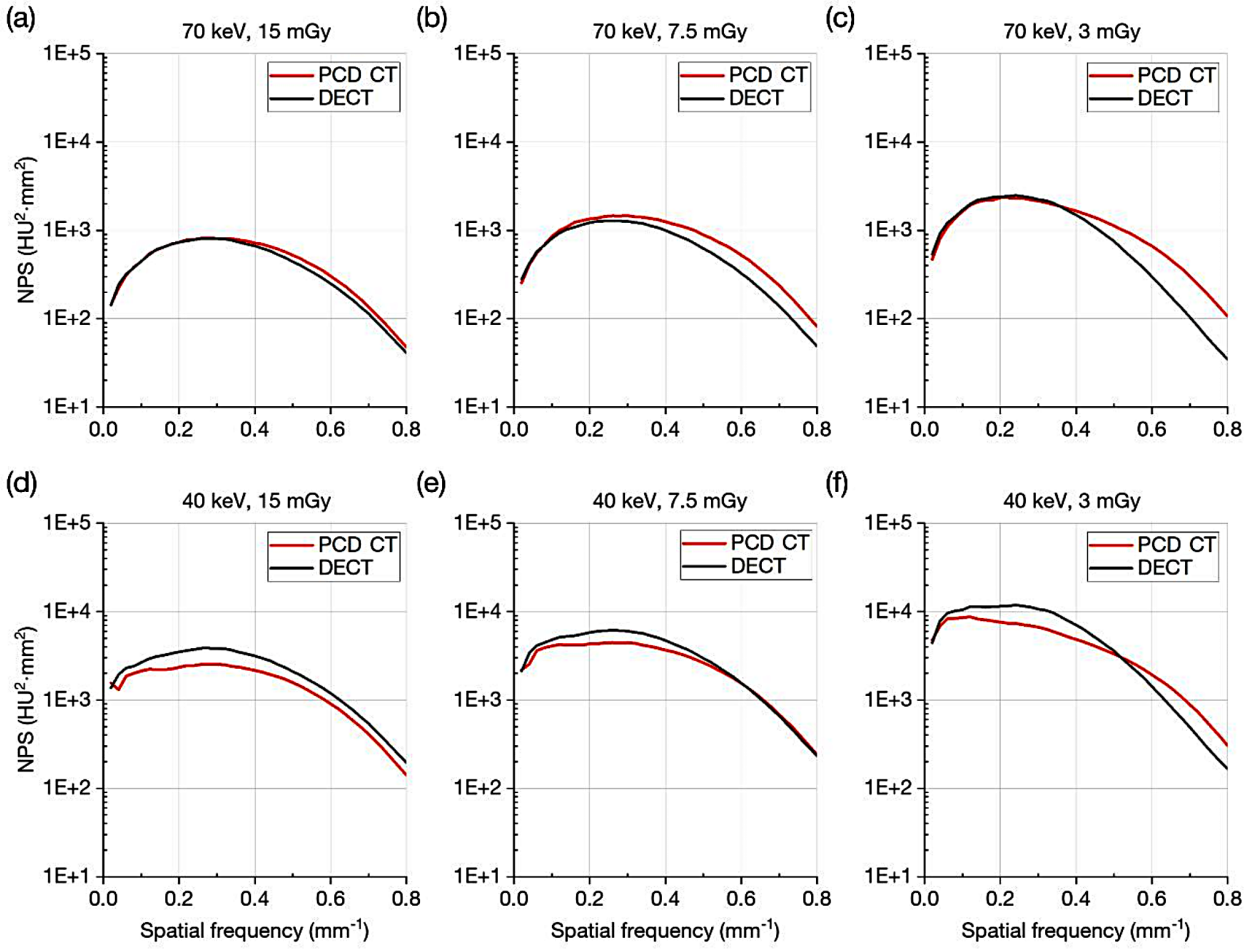
Comparison of noise power spectrum between photon-counting detector and dual energy CT. **a**, **b**, **c** 70 keV and **d**, **e**, **f** 40 keV at the volume CT dose indexes of **a**, **d** 15 mGy, **b**, **e** 7.5 mGy and **c**, **f** 3 mGy

The NPS results of PCD CT and DECT are shown in Fig. 3. For VMIs at 70 keV, the NPS values of PCD CT were almost identical to those of DECT at low spatial frequencies and somewhat higher at mid/high spatial frequencies. On the other hand, at 40 keV, the NPS values were lower with PCD CT than with DECT, except for the lowest frequency (i.e., 0.02 mm^− 1^) and high frequencies at 7.5 and 3 mGy. Focusing on the relationship between CTDI and NPS, the increase in high-frequency noise was suppressed in lower dose settings, especially for DECT. As a result, the NPS values at the high spatial frequency gradually became lower with DECT than with PCD CT as the CTDI values decreased.

### Slice sensitivity profile

The measured FWHMs of SSP are summarized in Table 2. Overall, FWHMs of PCD CT were smaller than those of DECT and closer to the nominal slice thickness of 1.0 mm. The ratios of FWHMs of DECT to those of PCD CT, which were used for the NPS correction, were 1.06, 1.14, and 1.10 for 15, 7.5, and 3 mGy, respectively, at 70 keV; 1.13, 1.32, and 1.26 for 15, 7.5, and 3 mGy, respectively, at 40 keV.

**Table 2:** Full width at half maximum of slice sensitivity profile of PCD-CT and DECT

	15mGy			7.5 mGy			3 mGy	
	PCD-CT	DECT		PCD-CT	DECT		PCD-CT	DECT
FWHM (mm)								
70 keV	1.31	1.38		1.34	1.52		1.51	1.67
40 keV	1.26	1.43		1.27	1.68		1.43	1.80

### Detectability index and system performance function

The *d’* values of PCD CT and DECT are summarized in Table 3. The *d’* values of PCD CT were comparable to those of DECT at 70 keV. The percentage increase was 12% or below. On the other hand, at 40 keV, PCD CT showed notably higher values by 25.7%, 34.4%, and 32.3% for 12 mgI/mL at 15, 7.5, and 3 mGy, respectively; 31.8%, 39.9%, and 28.3%, respectively, for 2 mgI/mL.

**Table 3:** Detectability index for 12 and 2 mg iodine/mL contrast task of PCD-CT and DECT

VM energy levels	Iodine contrast	15 mGy		7.5 mGy		3 mGy	
		PCD-CT	DECT	PCD-CT	DECT	PCD-CT	DECT
70 keV							
	12 mg/mL	64.3	61.2	47.3	43.1	33.3	29.8
	2 mg/mL	10.6	10.3	7.80	7.27	5.41	5.01
40 keV							
	12 mg/mL	90.1	71.7	65.2	48.5	42.7	32.3
	2 mg/mL	14.5	11.0	10.3	7.38	6.67	5.20

**Fig. 4:**
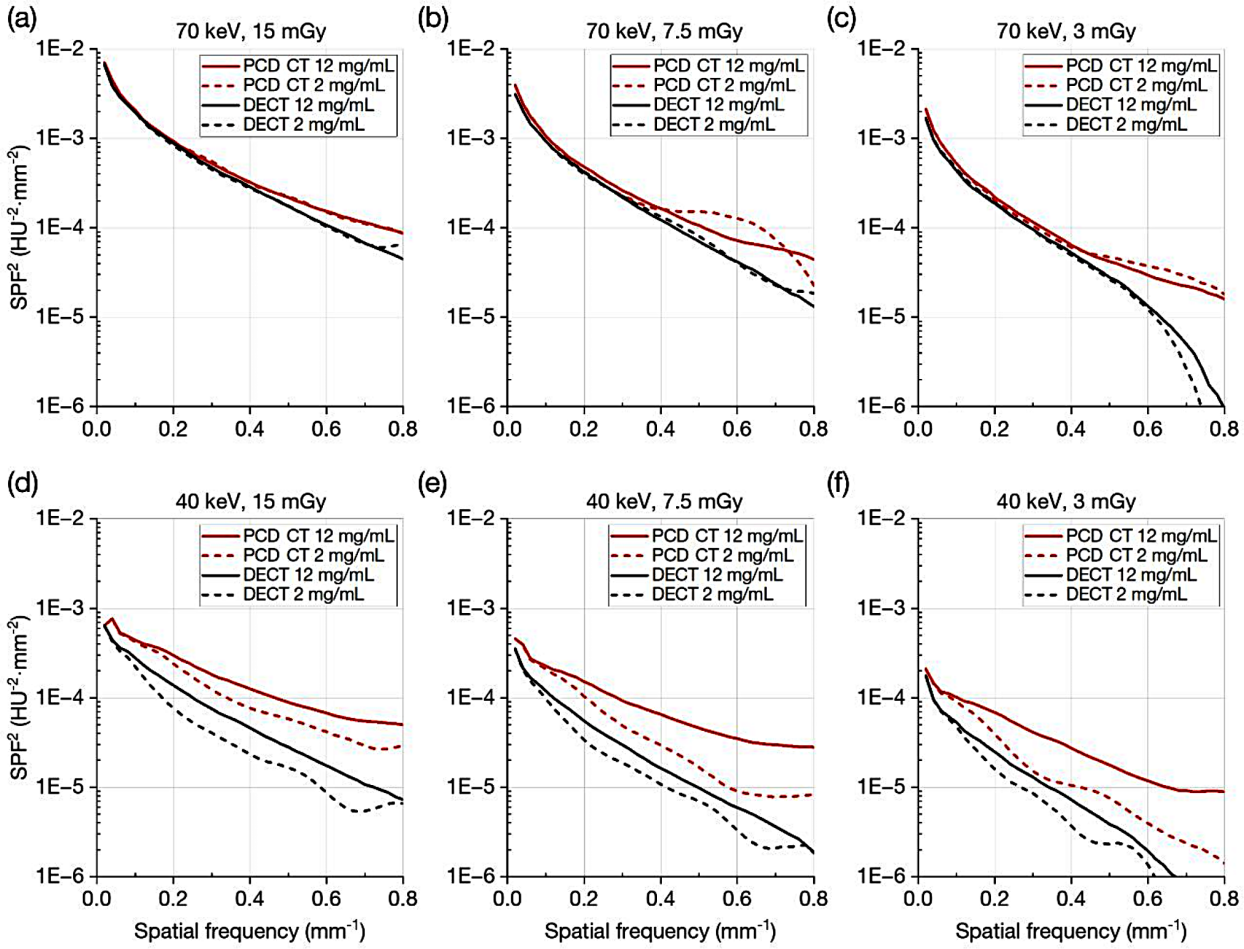
Comparison of squared system performance function between photon-counting detector and dual energy CT. **a**, **b**, **c** 70 keV and **d**, **e**, **f** 40 keV at the volume CT dose indexes of **a**, **d** 15 mGy, **b**, **e** 7.5 mGy and **c**, **f** 3 mGy

**Fig. 5:**
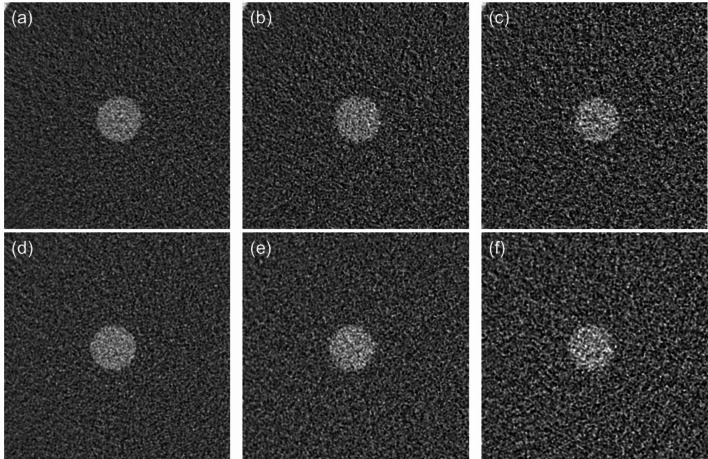
Comparison of virtual monoenergetic images at 70 keV of the rod with 2 mg iodine/mL. These images were obtained at volume CT dose indices of **a** 15 mGy, **b** 7.5 mGy, and **c** 3 mGy for PCD CT; **d** 15 mGy, **e** 7.5 mGy, and **f** 3 mGy for DECT. The window width/level was set at 250/30

**Fig. 6:**
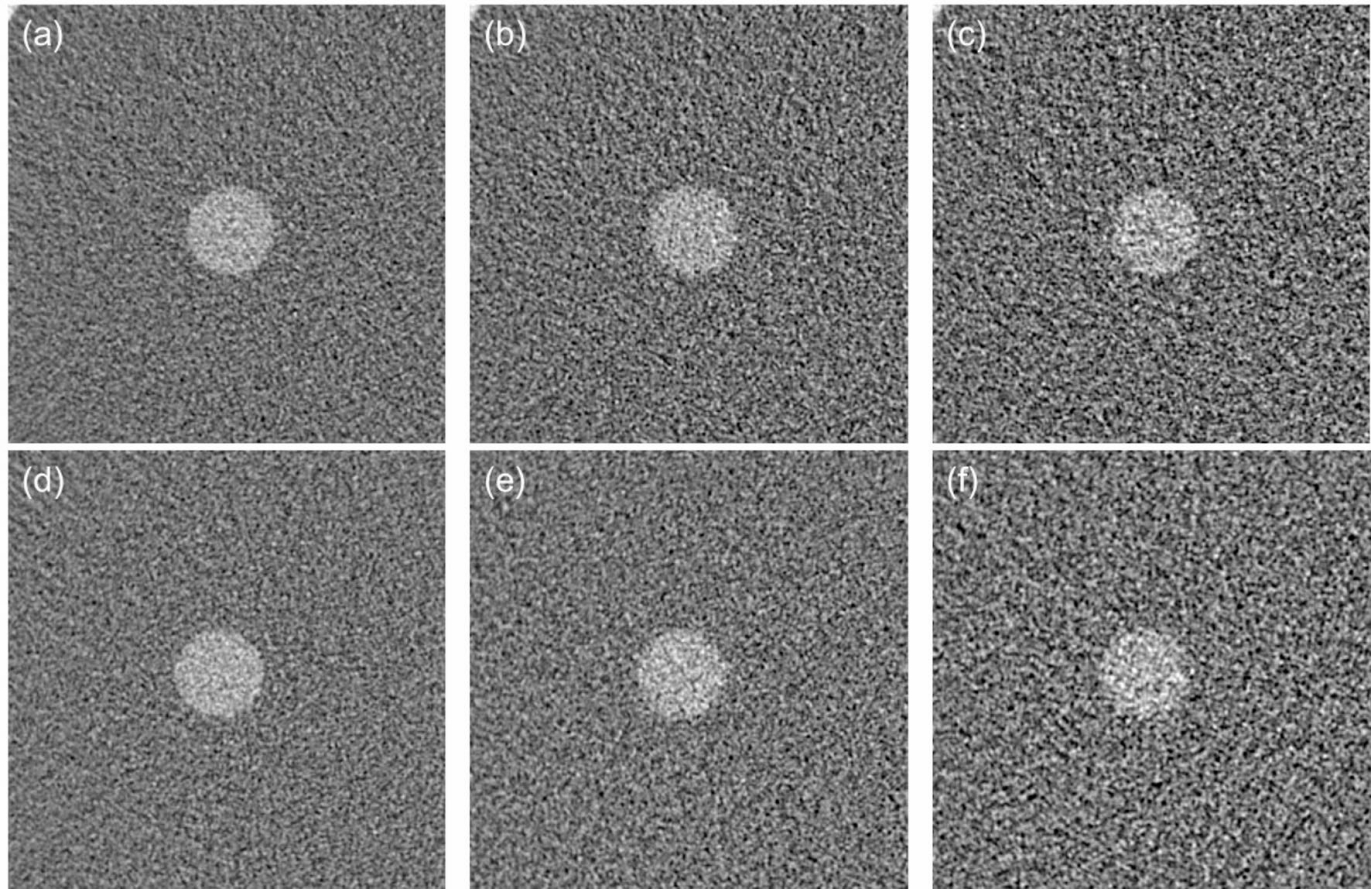
Comparison of virtual monoenergetic images at 40 keV of the rod with 2 mg iodine/mL. These images were obtained at the volume CT dose indices of **a** 15 mGy, **b** 7.5 mGy, and **c** 3 mGy for PCD CT; **d** 15 mGy, **e** 7.5 mGy, and **f** 3 mGy for DECT. The window width/level was set at 680/100

Figure 4 shows the SPF^2^ values. At 70 keV, though no performance differences between PCD CT and DECT were found in low spatial frequencies, the SPF^2^ values of PCD CT were somewhat higher than those of DECT at around more than 0.4 mm^− 1^. At 40 keV, PCD CT showed markedly improved performance for each iodine contrast task, except for the lowest spatial frequency (i.e., 0.02 mm^− 1^). The SPF^2^ values of PCD CT were higher than those of DECT as the spatial frequency increased. As shown in Figs. 5 and 6, the visual representation and the results of frequency-dependent characteristics show good correspondence. The VMIs at 70 keV turned out to be almost comparable between PCD CT and DECT. On the other hand, the VMIs at 40 keV in PCD CT showed less noise, and the edge of the 2 mgI/mL rod was clearly sharper than that in DECT.

## Discussion

With VMIs at 70 and 40 keV, we compared TTF, NPS, *d’* and SPF^2^ of PCD CT with those of DECT. These frequency-dependent characteristics of VMIs with different energy levels have not been investigated for the performance comparison between the clinical PCD CT and DECT. Our phantom study revealed that, at 40 keV, the *d’* values for iodine contrast tasks with a 5-mm diameter of PCD CT were notably higher than those of the DECT even with the low contrast task for the 2 mgI/ml concentration. Imaging performance estimated by SPF^2^ was remarkably higher with PCD CT than with DECT as the spatial frequency increased. These advantageous results of PCD CT were consistent with the rod phantom images, which showed sharper edges and lower noise. In contrast, at 70 keV, performance improvement was not so noticeable.

The superiority of PCD CT over DECT for TTF was pronounced at 40 keV. One possible reason for this is the equal weighting in low energy photon detections of PCD. In the low energy VMI reconstruction for DECT used in this study, a frequency split method was employed to suppress the noise enhanced by the process of low energy VMI generation. The resultant low energy images tended to have an inferior TTF because it was difficult to compensate for the edge structure at a low energy level (40 keV in this study) using the edge information of the mixed image [12, 18, 19, 39]. Although the reconstruction process of the 40 keV VMIs of PCD CT is unknown, it is conceivable that the low energy photons contribute more to the reconstruction for the 40 keV. As a result, at 40 keV, the edge information was more easily reproduced. In fact, the TTFs of PCD CT for the 12 mgI/ml at 40 keV were almost equal to those at 70 keV at 15 and 7.5 mGy. For the 2 mgI/ml, the decrease in TTF was less conspicuous in PCD CT than in DECT. It should be noted that these better TTFs at 40 keV were achieved without increasing NPSs as shown in Fig. 3d and f, which led to the higher *d’* values and SPF^2^.

For VMIs at 70 keV, the *d’* values of PCD CT for the iodine contrast task were comparable to those of DECT, while improvements in SPF^2^ of PCD CT were observed only at high spatial frequencies. This result is somewhat unexpected since the PCD does not require any septa between detector pixels, which are always required with the EID [1]. It would be natural to expect some improvements in geometric dose efficiency if septa are not required. The presumable reason for the non-improvement for PCD CT at 70 keV is the presence of an anti-scatter grid placed on the detector, which decrease dose efficiency due to the opacity walls forming them [1, 10]. When the wall positions match the septa in EID of DECT, the decrease in dose efficiency by the grids are mitigated. However, in PCD CT without septa, a decrease in dose efficiency by the grids is unavoidable; therefore, the PCD’s benefit thanks to “no septa” requirement seems to have been cancelled out.

Unlike at 70 keV, a dramatic performance improvement in PCD CT can be achieved with VMIs at 40 keV. Thanks to the absence of weighting reduction for low energy photons, the relative contribution of low-energy photons to the image formation process is higher than in the case of the EID [1, 9]. As a result, the edge information is maintained to a certain extent as shown in the TTF results, and also, noise increase, which is unavoidable in VMI reconstruction of DECT, is suppressed as shown in the NPS results. The superiority of PCD CT’s VMIs at low energies has been reported in CNR-based image quality evaluation studies [8, 9]. However, there are few reports on the benefits of the spatial frequency-dependent performance of VMIs in PCD CT. We found that the increased SPF^2^ values at higher spatial frequencies were reflected in the sharp visibility of the 2 mgI/mL rod edge. This means that VMIs at 40 keV in PCD CT have good potential use in iodine contrast enhancement without compromising image quality.

In the low dose settings (i.e., CTDI of 7.5 and 3 mGy), the percentage increase in the *d’* values of PCD CT tended to be somewhat higher than in the standard dose setting (15 mGy). It has been reported that, in low-dose settings, electronic noise from the detector system, which does not carry any diagnostic information and increases image noise, is more likely to affect the image quality [40]. With the PCD, it is easier to minimize such noise. We believe that our results support the superiority of the PCD CT at low dose settings, consistent with the previous report [2].

Our study has several limitations. First, only one size of the water phantom was used and the contents of the phantom were limited. It would be desirable to make the evaluation using a variety of sizes corresponding to different parts of both adult and pediatric bodies. Furthermore, in assessing the noise reduction technique of low energy VMIs in more detail, it would be worthwhile to use more sophisticated phantoms with non-uniform contents for simulating complicated structures or thin wires for simulating enhanced vessels, which show different physical image qualities than those obtained from rod phantoms [41, 42]. Second, we did not use IR to compare basic performance of PCD CT and DECT. Since IR is routinely used in clinical situations, performance comparisons for typical IR settings for PCD CT and DECT would be more practical. Third, we used only one tube voltage of 120 kV for PCD CT because it has a wide spectrum, which is generally preferable in spectral analysis in PCD CT. However, it has been reported that the iodine CNR was maximized in VMIs at 40 keV with a tube voltage of 90 kV in PCD CT [8]; thus, it is worthwhile to investigate how the *d’* and SPF^2^ are affected by different tube voltages. Fourth, the differences in effective slice thickness were corrected using the inverse square relationship between image noise and slice thickness. We confirmed that this relationship is maintained to some extent for small differences in slice thickness, but whether it holds for VMI over a wide range of energy and dose levels remains to be investigated. A more appropriate correction method for image comparisons with different slice thicknesses is a subject for future work.

## Conclusion

We evaluated VMI performance for the iodine contrast task at 70 and 40 keV with PCD CT and DECT, using frequency-dependent measurements. Our results demonstrated that VMIs in PCD CT were markedly superior to those in DECT at 40 keV, while their performance was comparable between PCD CT and DECT at 70 keV. The frequency-dependent results of the present study confirmed in more detail the previously reported superiority of PCD CT over DECT.
